# Racial disparities in chronic kidney diseases in the United States; a pressing public health challenge with social, behavioral and medical causes

**Published:** 2015-12-12

**Authors:** Shervin Assari

**Affiliations:** ^1^Department of Psychiatry, University of Michigan, Ann Arbor, MI, USA; ^2^Center for Research on Ethnicity, Culture and Health, School of Public Health, University of Michigan, Ann Arbor, MI, USA

**Keywords:** Health disparities, Chronic kidney disease, End stage renal disease, Socio-economics

**Implication for health policy/practice/research/medical education:**

In the United States, Blacks are at higher risk of morbidity and mortality associated with chronic kidney diseases compared to Whites. While only 13% of Americans are Black, 32% of kidney failures occur among Blacks. This problem becomes even more tragic given lower access of Blacks to kidney transplantation.

Compared to White Americans, Black Americans are at higher risk of morbidity and mortality associated with chronic kidney diseases (1,2). Blacks are at 3-4 times higher risk of developing kidney failure compared to Whites. While only one in eight Americans is Black, one-third of kidney failures happen among Blacks (1). These figures make inequality in morbidity and mortality attributed to kidney failure a major component of racial health disparities in the United States (3,4). This problem becomes even more tragic and challenging given lower access of Blacks with end-stage renal disease (ESRD) to treatment of choice of ESRD, being renal transplantation (5).

Similar to other aspects of health disparities, Black-White inequalities in chronic kidney diseases has complex and interwoven social, behavioral, and medical causes (1,2,4). Most proximal to the problem are disparities in chronic medical conditions that are proven risk factors for chronic kidney diseases (6,7). These include hypertension (8), diabetes (9), and obesity (10) that increase risk of end stage renal disease and are more known common among Blacks (11-14).

To better understand different aspects of Black-White inequalities in chronic kidney diseases in the United States, we recently conducted three studies. For all these studies, we borrowed Americans’ Changing Lives study (ACL) data, a nationally representative prospective cohort conducted from 1986 to 2011. The study included 3361 Black (n = 1156) or White (n = 2205) adults 25 and older who were followed for up to 25 years. Data was collected on race (as main predictor or moderator), baseline socio-economics, chronic medical disease (diabetes, hypertension, obesity), and health behaviors (smoking, drinking, and exercise) [as predictors, confounders, or mediators], and death due to renal disease over 25 years as the main outcome. Causes of death were extracted from death certificates or national death index, and were coded based on ICD-9 or ICD-10 codes, depending on the year of death. As the study has enrolled nationally representative sample, the results are generalizable to the US populations (15,16).

In the first study, in age and gender adjusted models, race was associated with risk of death due to renal disease over the follow up period. In separate models, socio-economics (income), health behaviors (smoking, drinking, and exercise) and chronic medical disease (diabetes and hypertension) fully explained the effect of race on mortality due to renal disease (15).

In two other studies, we compared Blacks and Whites for the predictive validity of factors that could potentially inform us about long term risk of deaths due to renal diseases in the general population. Based on one study, baseline depressive symptoms interacted with race, suggesting a weaker predictive role of baseline depressive symptoms on deaths due to renal diseases for Whites compared to Blacks. In race-specific models, high depressive symptoms at baseline only predicted risk of death due to renal diseases among Whites but not Blacks (2).

In the other study, Blacks and Whites where compared for effects of self-rated health (SRH) as a predictor of subsequent mortality due to kidney disease. The study showed Blacks and Whites differ regarding predictive role of baseline SRH on death due to renal diseases over time, with a weaker predictive role of poor SRH on deaths due to renal diseases for Whites compared to Blacks. Again, in race-specific models, SRH predicted risk of death due to renal diseases among Whites but not Blacks (16).

To conclude, first, Blacks are at higher risk of deaths due to renal disease. Second, such disparity is complex in nature and has multiple social, economic, behavioral, and medical (e.g. diabetes and hypertension) causes. Finally, it may be more difficult to separate Blacks who are at higher risk for subsequent death due to renal disease using some of the variables that can discriminate Whites who have higher risk. Lower predictive validity of some of the risk measures for Blacks means that targeted prevention programs will be more challenging to design for Blacks than Whites. These studies collectively extend the existing knowledge on racial disparities in renal disease and associated mortality. This is particularly important because Black-White disparity in chronic kidney disease morbidity and mortality is a major public health challenge in the United States (17).

## Author’ contribution

SA is the single author of the manuscript.

## Conflicts of interest

The author declared no competing interests.

## Ethical considerations

Ethical issues (including plagiarism, data fabrication, double publication) have been completely observed by the author.

## Funding/Support

No funding from any source is directly associated with this manuscript.
